# TOPK promotes lung cancer resistance to EGFR tyrosine kinase inhibitors by phosphorylating and activating c-Jun

**DOI:** 10.18632/oncotarget.6826

**Published:** 2016-01-06

**Authors:** Ying Li, Zhiwei Yang, Weijie Li, Shudi Xu, Tao Wang, Ting Wang, Mengjie Niu, Shengli Zhang, Lintao Jia, Shengqing Li

**Affiliations:** ^1^ Department of Pulmonary and Critical Care Medicine, Xijing Hospital, Fourth Military Medical University, Xi'an, China; ^2^ Department of Applied Physics, School of Science, Xi'an Jiaotong University, Xi'an, China; ^3^ State Key Laboratory of Cancer Biology, Department of Biochemistry and Molecular Biology, Fourth Military Medical University, Xi‘an, China; ^4^ Department of Neurology, Shaanxi Provincial People's Hospital, Xi'an, China; ^5^ Engineering Research Center of Forest Bio-preparation, Ministry of Education, Northeast Forestry University, Harbin, China; ^6^ Department of Pulmonary Medicine, Xi'an Ninth Hospital, Xi'an, China

**Keywords:** TOPK, lung cancer, EGFR-TKI resistance, c-Jun, AP-1

## Abstract

Tyrosine kinase inhibitors (TKIs) targeting the epidermal growth factor receptor (EGFR) have shown promising clinical efficacy in non-squamous non-small cell lung cancer (NSCLC); however, resistance is frequently observed in malignant cells, operating through a mechanism that remains largely unknown. The present study shows that T-lymphokine-activated killer cell-originated protein kinase (TOPK) is upregulated in NSCLC and excessively activated in TKI-refractory cells. TOPK dictates the responsiveness of lung cancers to the EGFR-targeted TKI gefitinib through the transcription factor AP-1 component c-Jun. TOPK binds directly to and phosphorylates c-Jun, which consequently activates the transcription of AP-1 target genes, including *CCND1* and *CDC2*. TOPK silencing sensitizes EGFR-TKI-resistant lung cancer cells to gefitinib and increases gefitinib efficacy in preclinical lung adenocarcinoma xenograft models. These findings represent a novel mechanism of lung cancer resistance to TKIs and suggest that TOPK may have value both as a predictive biomarker and as a therapeutic target: TOPK-targeted therapy may synergize with EGFR-targeted therapy in lung cancers.

## INTRODUCTION

Lung cancer with distal metastasis has an extremely high mortality rate [1]. Compared with the limited efficacy of traditional chemo- and radiotherapy in combination with surgery, molecular targeted chemicals, particularly EGFR tyrosine kinase inhibitors (TKIs), have emerged as potent anticancer agents and have significantly improved the prognosis of lung cancer patients with EGFR mutations [2]. Unfortunately, despite great progress in the treatment of lung cancer, resistance to EGFR-TKIs commonly occurs, leading to a modest response rate to first-line TKI therapy or to frequent recurrence after temporarily effective TKI treatment [3, 4]. Investigation of the molecular events that determine the response of lung cancer cells to TKIs is in progress to devise strategies to overcome resistance [5, 6].

Three molecular mechanisms of EGFR-TKI resistance are reported: the EGFR T790M gatekeeper mutation[2], activation of bypass signaling [7-9], and activation of downstream signaling [10, 11]. Activation of downstream signaling includes PI3K/AKT signaling activation, usually caused by loss of PTEN [10], and MAPK signaling activation, usually caused by ERK2 activation [11], EML4-ALK mutation [12], BRAF mutation [13] or KRAS mutation [14]. The present study found that upregulation of T-lymphokine-activated killer cell-originated protein kinase (TOPK), a serine/threonine protein kinase, is a new mechanism of resistance to gefitinib downstream of EGFR in NSCLC.

TOPK is frequently activated in several malignancies [15]. TOPK overexpression was detected in more than 60% of patients with lung cancer and correlates with poor disease-free survival [16]; TOPK also confers resistance of neoplastic cells to drug-induced apoptosis, suggesting that TOPK plays an important role in the occurrence and progression of cancers, probably by phosphorylating a wide range of substrate proteins [17, 18]. TOPK phosphorylates MAPKs, including the c-Jun N-terminal kinase (JNK), p38 and ERK, and interacts with p53 to repress transactivation of p21 during carcinogenesis [19, 20]. The present study reveals that TOPK is highly expressed in lung cancer but not in paraneoplastic tissues, and that excessive TOPK confers resistance of cancer cells to EGFR-TKIs.

The uncontrolled growth and division that is a hallmark of malignant cells originates from intensification of growth factor signaling, which ultimately activates key transcription factors and drives the expression of genes involved in biosynthesis, energy production and cell cycle progression [21]. The activator protein 1 (AP-1), which belongs to the class of basic leucine zipper (bZIP) transcription factors, plays essential roles in regulating genes involved in cell proliferation, survival, migration and transformation [22]. AP-1 is assembled by dimerization of Jun and Fos family members [22]. Of note is the c-Jun/c-Fos dimer, the overexpression or hyperactivation of which is implicated in diverse cancers [23]. Although the expression of c-Jun and c-Fos is regulated both at the transcriptional and translational levels, the activation of these proteins by posttranslational modification is a common mechanism that allows rapid alteration of gene expression in response to extracellular signals [22, 23]. For example, various extracellular stimuli can activate JNK, which subsequently phosphorylates and activates c-Jun for target gene transcription [22, 24]. Given the extensive involvement of c-Jun/c-Fos in gene expression and in diverse cellular processes, it is of great importance to decipher the mechanism of their activation in different cell signaling contexts. This study revealed that TOPK can activate AP-1 by directly binding to and phosphorylating c-Jun at serine 63 and 73, which consequently promotes the expression of a cohort of genes responsible for malignant cell division and survival.

## RESULTS

### TOPK expression correlates with TKI resistance in lung cancer cells

**Figure 1:**
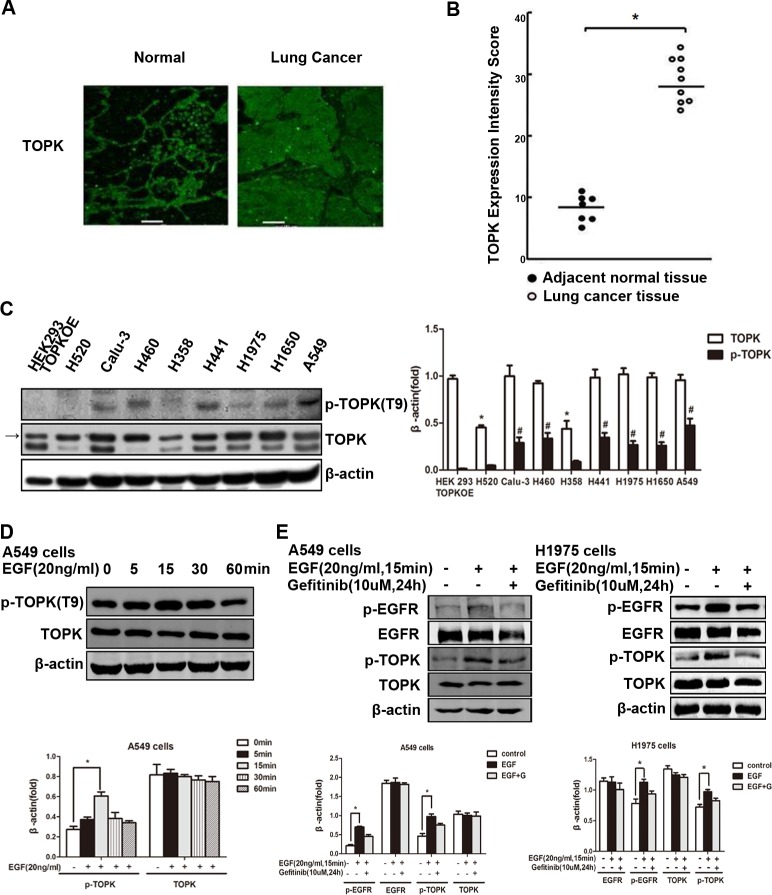
TOPK is overexpressed and activated in EGFR-TKI-resistant lung cancer cells **A.** Arrays of lung cancer tissues and paired normal adjacent tissues. The expression of TOPK was observed by laser scanning confocal microscopy. Representative staining is shown. **B.** TOPK expression intensity scores. *P < 0.05. **C.** TOPK expression levels in lung cancer cell lines. TOPK and phospho-TOPK were detected by Western blot analysis. HEK293 cells overexpressing TOPK (TOPKOE) were used as a positive control. Representative blots of 3 independent experiments were presented. Each bar represents the mean±SD from three experiments.*p < 0.05 compared with the TOPK level of HEK293 TOPKOE, ^#^*p* < 0.05 compared with the p-TOPK level of HEK293 TOPKOE. **D.** Western blot analysis of A549 lung cancer cells exposed to EGF (20 ng/mL) for indicated time. Representative blots of 3 independent experiments were presented. All protein levels were measured with densitometry and normalized to β-actin. Each bar represents the mean±SD from three experiments.*p < 0.05. **E.** Western blot analysis of lung cancer cells exposed to EGF (20 ng/mL) for 15 min after addition of gefitinib (10 μM) for 24 h. Representative blots of 3 independent experiments were presented. All protein levels were measured with densitometry and normalized to β-actin. Each bar represents the mean±SD from three experiments.*p < 0.05 *vs* control.

To investigate the role of TOPK in the development and progression of lung carcinomas, we first compared the expression of TOPK in clinical specimens of lung cancer and adjacent normal tissue, and detected significant upregulation in malignant tissues compared to normal tissues (Figure 1A, 1B). We next measured TOPK levels in lung cancer cell lines with varied sensitivity to EGFR-TKIs. We found that TOPK was overexpressed in EGFR-TKI-resistant lines (Figure 1C, 1D), including calu-3, H460, H441, H1975, H1650 and A549. TOPK expression was much lower in EGFR-TKI responsive cell lines, including H520 and H358. In addition, TOPK activation, assessed by phospho-TOPK, was also much higher in the resistant lines than in the responsive lines (Figure 1C, 1D), suggesting that TOPK may play a role in TKI resistance. One of the molecular mechanisms of EGFR-TKI resistance is activation of signaling downstream of EGFR. In this study, we found in both A549 and H1975 cells that EGFR activation stimulated by EGF can be blocked by the EGFR-TKI gefitinib; however, TOPK phosphorylation occurred downstream of EGF/EGFR and was only partially inhibited by gefitinib (Figure 1E). Therefore, excessive TOPK expression and activity correlates with resistance to EGFR-TKIs in lung cancer cells.

**Figure 2:**
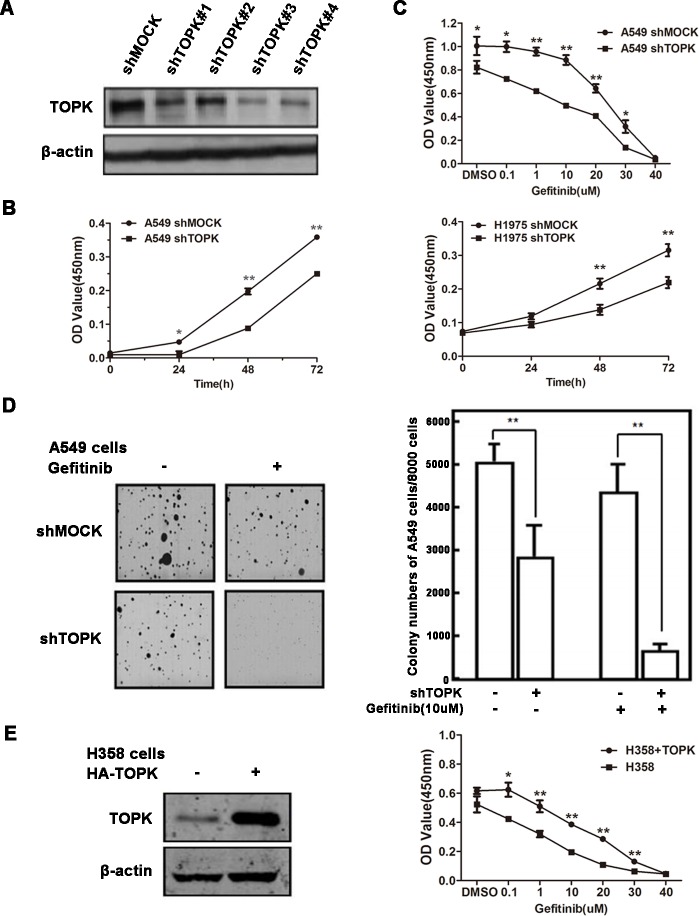
TOPK desensitizes lung cancer cells to gefitinib **A.** Knockdown of TOPK in A549 cells. A549 cells were infected with control lentiviral particles (shmock) and four different TOPK-targeted lentiviral particles (shTOPK). TOPK protein levels were detected by Western blot. The most efficient TOPK knockdown cell line (A549-shTOPK#3) was used for further study. **B.** Knockdown of TOPK inhibits A549 and H1975 cell growth. Cell proliferation assay following infection with lentiviruses expressing mock or TOPK-target shRNAs. **C.** Knockdown of TOPK increases the sensitivity of A549 cells to gefitinib in cytotoxicity assays. Cells expressing the indicated shRNAs were exposed to gefitinib for 48 h. **D.** Knockdown of TOPK increases the sensitivity of A549 cells to gefitinib in anchorage-independent growth assays. Cells were exposed to 10 μM gefitinib. Colonies were counted using a microscope and the Image-Pro Plus software (v4). Representative photographs are shown. **E.** Ectopic expression of TOPK in H358 cells makes cells resistant to gefitinib. Cells were transiently transfected with pcDNA3.1(+)-TOPK or pcDNA3.1(+). The cells were cultured for 24 h, and then proteins were extracted for TOPK expression analysis (left). Cell growth was measured by cytotoxicity assay after exposure to gefitinib for 48 h. The data are shown as the means ± SDs of triplicate samples. The asterisk (*) indicates a significant decrease (*P* < 0.05), and the double asterisk (**) indicates a significant difference (*P* < 0.01) compared to control.

We next examined whether TOPK directly affected the sensitivity of lung cancer cells to EGFR-TKIs. TOPK was knocked down in lung cancer cells by short hairpin RNAs (shRNAs) (Figure 2A). TOPK silencing significantly inhibited the growth of both A549 and H1975 cells, which were known to be refractory to EGFR-TKI treatment (Figure 2B) [25, 26]. TOPK knockdown enhanced gefitinib-induced inhibition of A549 cell growth and colony formation (Figure 2C & 2D). Conversely, ectopic expression of TOPK in a TKI-sensitive lung cancer cell line, H358, decreased the responsiveness to gefitinib (Figure 2E) [25]. These data suggest that TOPK plays an essential role in regulating the sensitivity of lung cancer cells to EGFR-TKIs.

### Molecular modeling suggests that TOPK interacts with c-Jun

**Figure 3:**
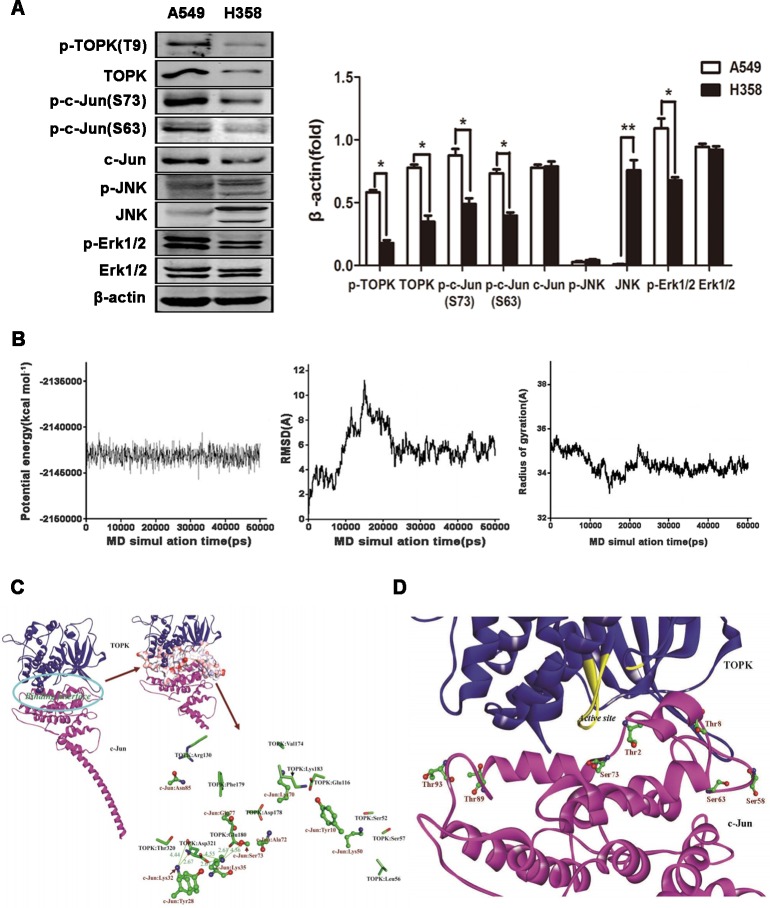
TOPK interacts with c-Jun in modeling studies **A.** c-Jun is activated in EGFR-TKI-resistant A549 cells. Western blot analysis of EGFR-TKI-resistant (A549 cells) and -responsive (H358 cells) lung cancer cells. β-actin was detected to assess protein loading. A representative blot was presented. All protein levels were measured with densitometry and normalized to β-actin. Each bar represents the mean±SD from three experiments.*p < 0.05, **p < 0.01*vs* H358. **B.** Time-evolution potential energy, backbone-atom RMSD and radius of gyration for the TOPK-c-Jun complexes. **C.** Propeller structure and key residues within the binding interface of the TOPK-c-Jun complex. The key residues of TOPK and c-Jun are represented using the stick and ball-and-stick models, respectively. Salt bridges are shown in olive green with distance values (unit in Å). **D.** Probable TOPK phosphorylation sites in c-Jun. Phosphorylation sites are represented using the ball-and-stick model.

To dissect the signaling downstream of TOPK responsible for cancer cell survival and division, we assessed the activation of potential TOPK substrate proteins, including ERK, JNK and c-Jun in EGFR-TKI-resistant (A549 cells) and -responsive (H358 cells) lung cancer cells [25]. Since TOPK and ERK phosphorylate each other upon stimulation by EGF [27], elevated phosphorylation of TOPK is accompanied by high-level ERK phosphorylation in A549 cells (Figure 3A, 3B). Unexpectedly, a significantly high level of phosphorylated c-Jun, but not of its classical activator phospho-JNK, was detected in EGFR-TKI-resistant cells, suggesting that c-Jun is not activated by JNK in EGFR-TKI-resistant cells (Figure 3A) but may be induced by TOPK either via direct interaction or via an intermediate kinase [16].

The structures of TOPK and c-Jun have not been characterized, hindering insights into regulatory mechanisms and future rational drug design. To study the interaction between TOPK and c-Jun, both protein structures were first modeled by homology. The two models were sufficiently equilibrated and refined within 10 ns, with good stereochemical features (Figures S1 and S2), to be suitable to study interactions. The TOPK-c-Jun complex reached equilibrium at approximately 30 ns (Figure 3B). Hence, the structural analysis is based on the average structure of the 30.0–50.0 ns MD trajectories. The binding of TOPK with c-Jun is characterized by strong electrostatic interaction networks, involving residues TOPK:Glu180, c-Jun:Lys35, TOPK:Asp321 and c-Jun:Lys32, with the formation of six salt bridges (Figure 3C). Seventeen H-bonds within the interfacial surfaces stabilize the complex (Suppl. Table 1). In addition, the polarity of the interface between TOPK and c-Jun was assessed. Polar residues, especially Lys, Ser and Glu, are likely to appear at the binding interface in high presence ratios (12.31, 10.77 and 9.23 %); Lys and Ser, in particular, are likely to make significant contributions to binding (Suppl. Table 1). In accordance with our binding model, seven c-Jun phosphorylation sites, residues Thr2, Thr8, Ser58, Ser63, Ser73, Thr89 and Thr93, are covered by TOPK and may be phosphorylated (Figure 3D). These modeling results inform on the molecular interaction between TOPK and c-Jun and may help shed light on the biological roles of TOPK, but require validation by immunoprecipitation and *in vitro* kinase assay.

### TOPK directly binds to and phosphorylates c-Jun in lung cancer cells

**Figure 4:**
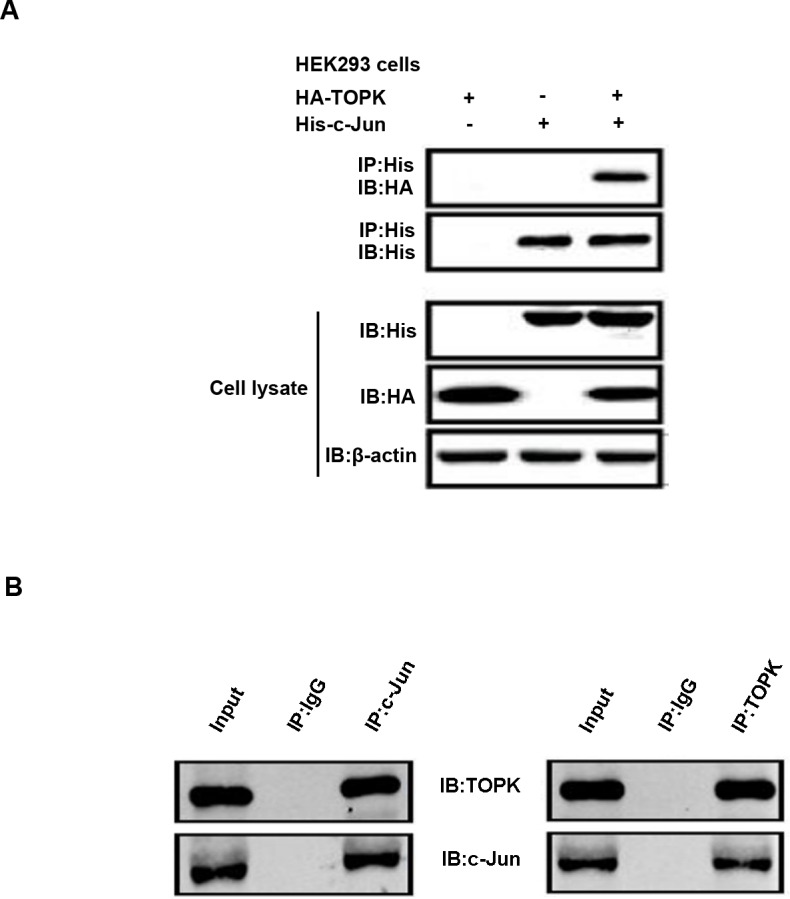
TOPK binds directly to c-Jun in lung cancer cells **A.** HEK293 cells were transfected with constructs expressing HA-TOPK and/or His-c-Jun, after which c-jun was immunoprecipitated with anti-His antibody and immunoblot analysis was conducted with anti-HA and anti-His antibodies. β-actin was detected to assess protein loading. **B.** Immunoprecipitation of A549 cell lysates (2mg) with anti-TOPK or anti-c-Jun antibodies was followed by Western blot analysis with anti-c-Jun or anti-TOPK antibodies. The loading quantity of input is 0.7mg.

The predicted three-dimensional structures of TOPK and c-Jun support a direct interaction model that requires cell-based validation. To test whether TOPK associates with c-Jun directly, we co-transfected HEK293 cells with HA-tagged TOPK and His-conjugated c-Jun. Immunoprecipitation assays showed that the ectopically expressed TOPK and c-Jun indeed interacted with each other (Figure 4A). Similarly, endogenous TOPK co-immunoprecipitated with c-Jun in A549 cells, suggesting that the two proteins could form a complex in EGFR-TKI-resistant lung cancer cells (Figure 4B).

**Figure 5:**
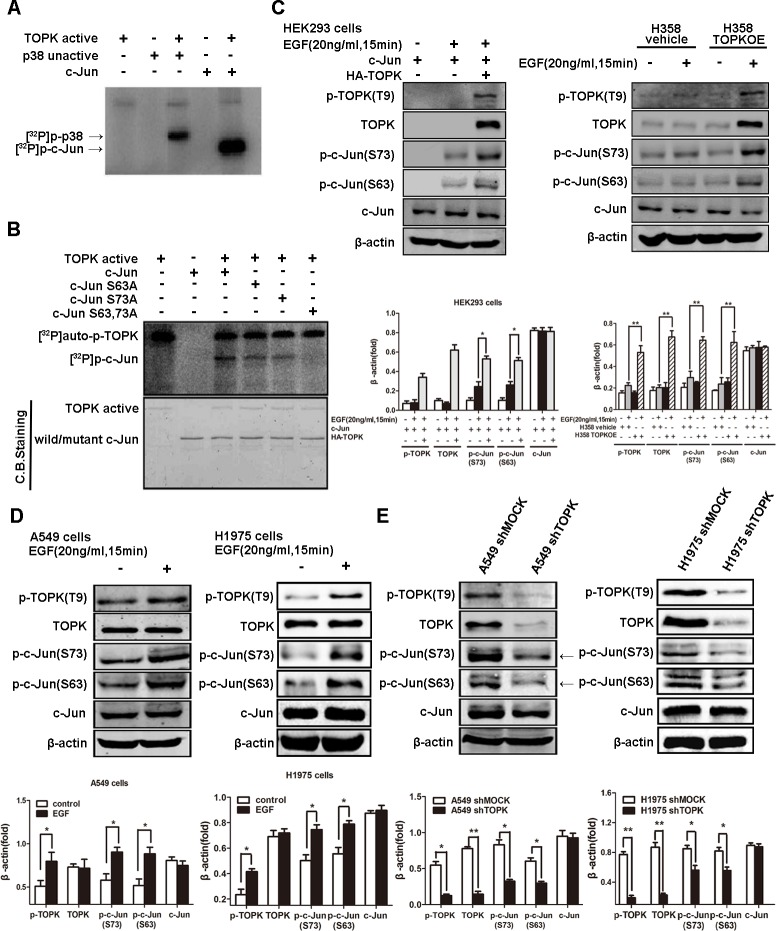
TOPK phosphorylates c-Jun at serine 63 and serine 73 **A.** Active TOPK phosphorylates full-length c-Jun *in vitro* in the presence of [γ-^32^P] ATP. The incorporation of ^32^P was visualized by autoradiography. Inactive p38 protein was used as a positive control. **B.** Active TOPK phosphorylation of wild-type c-Jun, c-Jun(S63A), c-Jun(S73A) and c-Jun (S63A&S73A) was visualized by autoradiography. Equal protein loading was visualized by Coomassie Blue (CB) staining. **C.** Ectopic expression of TOPK increases c-Jun phosphorylation at the Ser63 and Ser73 sites. Cells were transfected with pcDNA3.1A-HA-TOPK and pcDNA4-His-c-Jun and cultured for 24 h. After starvation in DMEM supplemented with 0.1% FBS for 24 h, cells were stimulated with EGF (20 ng/mL) and harvested 15 min later. Whole cell lysates were then analyzed by Western blotting. A representative blot was presented. All protein levels were measured with densitometry and normalized to β-actin. Each bar represents the mean±SD from three experiments.*p < 0.05, **p < 0.01. **D.** EGF induces TOPK activation and c-Jun phosphorylation at Ser63 and Ser73 in gefitinib-resistant lung cancer cells. After starvation in RPMI 1640 supplemented with 0.1% FBS for 24 h, cells were stimulated with EGF (20 ng/mL) and harvested 15 min later. Whole cell lysates were then analyzed by Western blotting. All protein levels were measured with densitometry and normalized to β-actin. Each bar represents the mean±SD from three experiments.*p < 0.05, **p < 0.01. **E.** Knockdown of TOPK decreases the phosphorylation level of c-Jun. The whole cell lysates were analyzed by Western blotting. A representative blot was presented. All protein levels were measured with densitometry and normalized to β-actin. Each bar represents the mean±SD from three experiments.*p < 0.05, **p < 0.01.

We next examined whether the direct binding of TOPK to c-Jun accounted for c-Jun phosphorylation. Indeed, an *in vitro* kinase assay indicated that radioactive phosphorus (^32^P) was incorporated into c-Jun by active TOPK, suggesting that TOPK catalyzes the specific phosphorylation of c-Jun (Figure 5A). To identify the phosphorylation sites, we mutated candidate residues, i.e., S63 or S73, by substituting alanine. Mutation of either serine attenuated c-Jun phosphorylation, whereas mutation of both decreased c-Jun phosphorylation dramatically (Figure 5B). Further, we found that the levels of c-Jun phosphorylation at both sites (S63 & S73) were increased in EGFR-TKI-resistant lung cancer cells compared to -responsive cells, supporting the involvement of TOPK-mediated c-Jun phosphorylation in EGFR-TKI resistance (Figure 3A). In addition, EGF stimulation resulted in simultaneous TOPK and c-Jun phosphorylation, both in HEK293 cells and H358 cells that expressed ectopic TOPK (Figure 5C) and in EGFR-TKI-resistant A549 and H1975 lung cancer cells that expressed high endogenous TOPK (Figure 5D). However, the phosphorylation level of c-Jun decreased dramatically in TOPK knockdown compared with the parental A549 cells (Figure 5E). These data confirm the phosphorylation of c-Jun by TOPK at serine 63 and 73 during the development of resistance to EGFR-targeted TKIs.

### TOPK phosphorylation of c-Jun induces the transcriptional activity of AP-1

**Figure 6:**
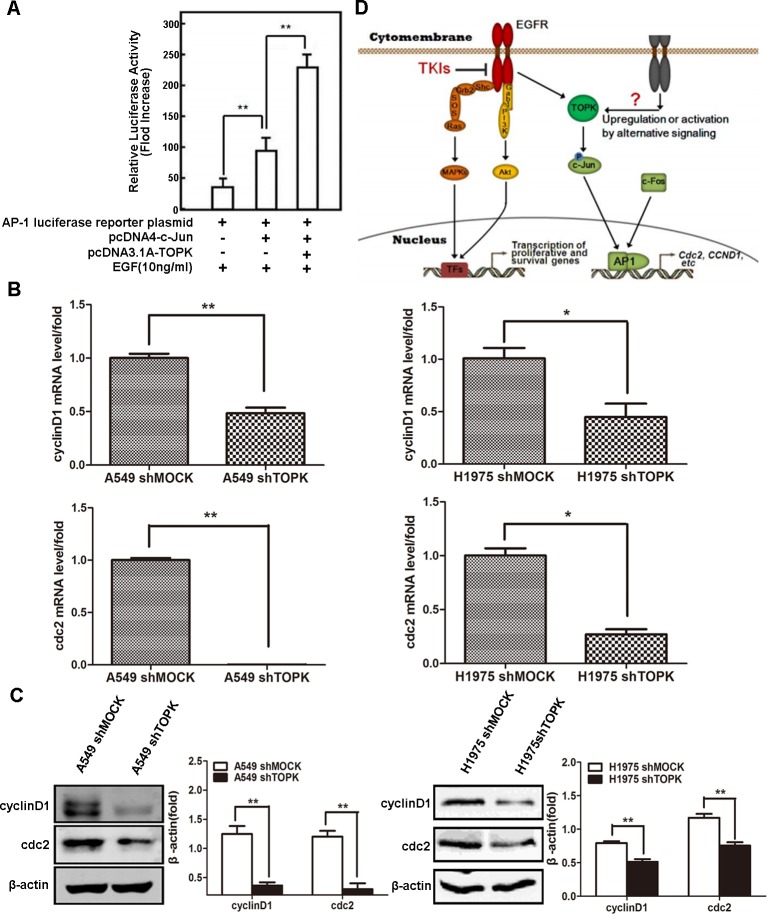
c-Jun phosphorylation by TOPK induces the transcriptional activity of AP-1 **A.** AP-1 transcriptional activity assay. HEK293 cells were transfected with AP-1 luciferase reporter plasmid, pcDNA3.1A-HA-TOPK and pcDNA4-His-c-Jun. Cells were starved in 0.1% FBS-DMEM for 24 h, after which they were stimulated with EGF (10 ng/mL) for 12 h. Then, the cells were disrupted in lysis buffer and luciferase activity was measured in a luminometer. **B.** *CCND1* and *CDC2* mRNA expression levels detected by qRT-PCR. Total RNA was extracted from cells for qRT-PCR analysis. β-actin was detected to assess loading. The data are shown as the mean ± SD of triplicate samples. *p < 0.05, **p < 0.01. **C.** Protein levels of cyclin D1 and Cdc2 measured by Western blot. The total proteins of cells modified with a control or shTOPK lentiviral construct were extracted for Western blot analysis. β-actin was detected to assess loading. All proteins were level measured with densitometry and normalized to β-actin. Each bar represents the mean±SD from three experiments. **p < 0.01. **D.** Schematic relationship of TOPK with EGF/EGFR signaling pathway. EGF stimulates membrane receptor EGFR and then activates MAPKs and PI3K/Akt to promote the transcription of cell proliferation and survival genes. However, in some EGFR-resistant cell lines, TOPK is activated downstream of EGF/EGFR signaling pathway, which can phosphorylate c-Jun and then increase AP-1 activity to promote the transcription of AP-1 target genes, e.g. *CCND1* and *CDC2*, etc.

The transcriptional activity of AP-1 is regulated by the phosphorylation of its components, e.g., c-Jun and c-Fos [24]. To test whether TOPK phosphorylation of c-Jun affects AP-1 transcriptional activity, we generated an AP-1 luciferase reporter construct and detected abundant luciferase expression in HEK293 cells co-expressing c-Jun and TOPK upon exposure to EGF (Figure 6A). Similarly, the mRNA expression levels of *CCND1* and *CDC2*, both of which are known AP-1 target genes, were significantly decreased in TOPK knockdown compared to the parental lung cancer cells (Figure 6B). The protein expression levels of cyclin D1 and Cdc2 were also significantly decreased after TOPK silencing in lung cancer cells exposed to EGF (Figure 6C). These results reveal that TOPK phosphorylation of c-Jun promotes the transcriptional activity of AP-1 in lung cancer cells, thereby promoting lung cancer cell division and proliferation (Figure 6D).

### TOPK promotes the resistance of lung adenocarcinoma to EGFR-TKI treatment *in vivo*

**Figure 7:**
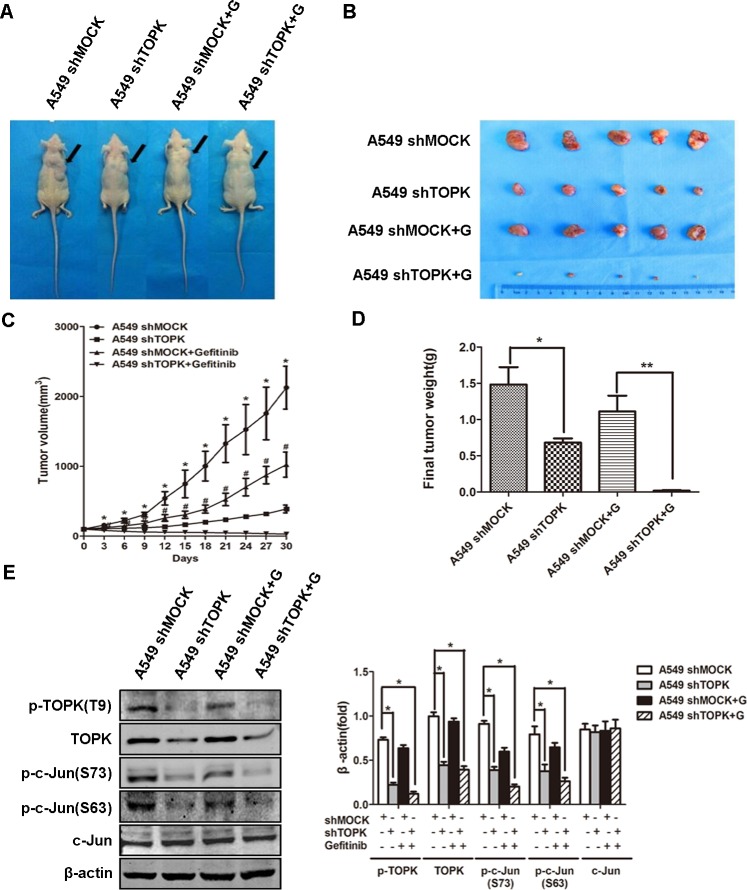
Knockdown of TOPK increases the responsiveness of human xenograft lung adenocarcinoma to gefitinib **A.** Nude mice bearing human xenograft A549-shmock and A549-shTOPK lung adenocarcinomas were treated with gefitinib (+ G) by oral gavage (25 mg/kg/d, *n* = 5) for 30 days. Mice were sacrificed and tumors were dissected 30 days after the first injection of gefitinib. **B.** Representative photographs of four groups of tumor-bearing mice at day 30. **C.** Tumor volume changes were recorded every 3 days from the first day of gefitinib treatment. The data are shown as means ± SDs (*n* = 5). The asterisk (*) indicates a significant difference (*P* < 0.05) between the A549-shTOPK group and the A549-shmock group, and the marker (#) indicates a significant difference (*P* < 0.05) between the A549-shTOPK + gefitinib group and the A549-shmock + gefitinib group. **D.** Tumors were weighed at day 30. The data are shown as means ± SDs (*n* = 5). *p < 0.05, **p < 0.01. **E.** Tumors were homogenized and assessed by Western blot for TOPK and c-Jun expression. A representative blot was presented. All the protein level measured with densitometry and normalized to β-actin. Each bar represents the mean±SD from three experiments. *p < 0.05.

We evaluated the role of TOPK in driving EGFR-TKI resistance in xenograft lung cancer mouse models. Nude mice were injected with A549 cells modified with a control construct or modified to express a TOPK-targeted shRNA to allow the formation of subcutaneous tumors. The mice were subsequently treated with gefitinib (25 mg/kg/d) by oral gavage for 30 days. Compared to gefitinib alone, gefitinib in combination with TOPK knockdown showed significantly increased suppression of tumor growth (Figure 7A, 7B). Gefitinib with TOPK knockdown also dramatically decreased tumor volume and tumor weight compared to gefitinib alone (Figure 7C, 7D). Further, TOPK knockdown caused a significant decrease in c-Jun phosphorylation in the xenograft tumor cells, which is consistent with the *in vitro* finding of c-Jun phosphorylation by TOPK (Figure 7E). These results indicate that TOPK plays an essential role in the responsiveness of lung cancers to EGFR-TKIs *in vivo*.

## DISCUSSION

Cell behaviors, including malignant transformation and drug resistance, are governed by intracellular signals originating from either outside or inside the cell [28]. Protein kinases, e.g., receptor tyrosine kinases (RTKs) that couple extracellular growth stimuli with intracellular signal transduction, play key roles in mediating signals that culminate in cell growth and division [29]. EGFR, which is frequently overexpressed or constitutively activated upon mutation, triggers the occurrence and progression of lung cancers [2]. Consequently, a class of TKIs targeting EGFR has emerged and contributed critically to clinical therapy in pulmonary malignancies [4]. The benefit of these drugs is limited by the fact that most patients inevitably develop resistance, i.e., machineries that circumvent the cytotoxicity of these EGFR-TKIs [30, 31]. Although the mechanisms underlying TKI resistance in lung cancers are diverse, the activation of downstream signaling pathways that converge with the EGFR pathway to activate the transcription of proliferation-related genes represents the predominant molecular event in EGFR-TKI-resistant neoplastic cells [5, 30, 31]. Indeed, we found that TOPK was significantly upregulated and profoundly activated in lung cancer cells that exhibited resistance to EGFR-TKIs. The activation of EGFR with the treatment of EGF in lung cancer cells was positively correlated with the phosphorylation of TOPK. TOPK phosphorylates and inactivates PTEN, which in turn activates Akt that leads to proper G2/M progression [32]. TOPK/PBK promotes cell migration via phosphorylating AKT at Ser (473) and decreasing PTEN levels. LY294002, a PI3K inhibitor, did not inhibit the TOPK-induced decrease in PTEN. These results indicate that the TOPK-mediated PTEN decrease has an upstream role in regulating PI3K/AKT-stimulated migration [33]. With EGF treatment, TOPK and ERK2 can phosphorylate each other, and lead to a positive feedback loop between TOPK and ERK2 in colorectal cancer [27].

Whereas TOPK can be activated by EGFR signaling, the insufficiency of EGFR-targeted TKIs to abrogate TOPK activation implies either the redundancy of TOPK with upstream EGFR signaling elements or the existence of alternative signaling pathways that elicit the phosphorylation and activation of TOPK. Actually, TOPK is upregulated upon activation of insulin-like growth factor 1 (IGF1) signaling [34]. It is reported to be the target gene of Hippo-YAP signaling [35] and EWS-FLI1pathway [36]. Expression of TOPK is regulated by cell cycle-specific transcription factors c-Myc, E2F [37] and CREB/ATF [38]. CDC2/cyclin B1 can inactivate protein phosphatase 1 alpha (PP1alpha) and caused enhancement of autophosphorylation of TOPK and resulted in its activation at an early stage of mitosis [39], so this is a positive feedback between TOPK and CDC2 according to our study.

The activator protein 1 (AP-1), predominantly composed of c-Jun and c-Fos, plays a critical role in the transcriptional regulation of a class of genes involved in cell survival, proliferation and differentiation [22, 24]. Overexpression of c-Jun and c-Fos underlies the development of various carcinomas, including pulmonary malignancies [22, 40], and commonly occurs in concert with the activation or stabilization of c-Jun by JNK, ERK and RhoC in response to various extracellular stimuli, including cytokines, growth factors and adhesion molecules, respectively [24]. Rather than through these canonical signaling pathways that regulate c-Jun, we established here that c-Jun is directly phosphorylated and activated by TOPK in EGFR-TKI-refractory lung cancer cells, leading to the transcriptional activation of AP-1 target genes such as *CCND1* and *CDC2* [41, 42]. Consequently, they may counteract TKIs by relieving cell cycle arrest induced by attenuated EGFR signaling. These findings are consistent with previous reports that *CCND1* and *CDC2* are highly expressed in lung adenocarcinoma, and that CDC2 is reported to predict bad prognosis in advanced NSCLC and may be a therapeutic target for advanced NSCLC patients [40, 43, 44]. In light of a previous report showing that the EGFR signaling pathway can promote AP-1 transcriptional activity [45], TOPK-induced activation of AP-1 may represent a critical mechanism to maintain AP-1 transcriptional activity upon suppression of EGFR activity by TKIs. While the levels of phosphorylated JNK were similar and very low among the TKI-sensitive and -resistant lung cancer cell lines tested, our study does not exclude the possibility of AP-1 regulation by ERK1/2. ERK2 and TOPK phosphorylate each other upon exposure to EGF in colorectal cancer [25]. We also found that in EGFR-TKI-resistant A549 cells, elevated phosphorylation of TOPK was accompanied by high-level ERK1/2 phosphorylation compared to EGFR-TKI-sensitive H358 cells. This means that ERK1/2 may also be involved in driving EGFR-TKI resistance in lung cancer.

TOPK correlates with cell transformation and poor prognosis in various malignancies [15]. TOPK also confers resistance to chemotherapy and to proapoptotic ligands such as TRAIL [17, 18]. The multifaceted functions of TOPK are reflected in the growing list of known TOPK partners and substrates including p53, ERK2, peroxiredoxin 1 (Prx1) and the p38α-specific phosphatase MKP1 [20, 27, 46, 47]. TOPK maintains NF-κB activity by phosphorylating IκBα, which is implicated in the resistance of cervical cancer to doxorubicin [17]. Intriguingly, TOPK also binds to and phosphorylates JNK1, a classical regulator of c-Jun activity [48]; however, it is unlikely in our study that TOPK activates c-Jun by phosphorylating JNK, given that the levels of total JNK and phospho-JNK were very low among the EGFR-TKI-sensitive and -resistant cell lines. The modeling studies supported a direct association between TOPK and c-Jun. Further studies confirmed interaction by showing that TOPK coimmunoprecipitates with c-Jun in A549 cells, and that c-Jun was phosphorylated by TOPK in an *in vitro* kinase assay. However, further investigation is needed to determine whether other proteins, e.g., transcriptional factors or the aforementioned TOPK substrates, are also involved in the TOPK-mediated TKI resistance of lung cancers. The systematic analysis of gene profiles affected by TOPK and c-Jun will provide a clear depiction of the integrated regulatory network underlying lung cancer resistance to EGFR-TKIs.

Accumulating evidence suggests that EGFR-TKI resistance arises from diverse genetic alterations and is characterized by a malignant cell population with apparent heterogeneity [31]. The EGFR mutant T790M, found in lung cancers with very high prevalence, can circumvent suppression by TKIs [49]. Gene amplifications or mutations that cause alternative growth factor receptor signaling, e.g., signaling downstream of the human epidermal growth factor receptor (HER) family, hepatocyte growth factor (HGF)/c-Met or insulin-like growth factor 1 receptor (IGF1R), confer resistance of cancer cells to EGFR-targeted TKIs [30, 31]. Mutations resulting in constitutively active kinases or transcriptional factors such as anaplastic lymphoma kinase (ALK), PI3K, MAPK and JAK/STATs, or mutations altering the levels of cell survival regulators, e.g., the pro-apoptotic protein BIM, also underlie the acquisition of TKI resistance [30, 31, 50]. Although we found that TOPK alone is sufficient to desensitize lung cancer cells to TKIs, extensive crosstalk between TOPK and the aforementioned canonical machinery of EGFR-TKI resistance may also exist [16, 30]. To this end, genome-wide screening of TKI-resistant neoplastic cells will help identify the genetic variations underlying TOPK overexpression and activation, and will help determine whether TOPK acts as a driver or mediator of EGFR-TKI resistance in human lung carcinomas [51]. Nevertheless, our study provides novel insights into the mechanism of TKI resistance, and thus suggests that TOPK may be a suitable therapeutic target for overcoming EGFR-TKI resistance in lung carcinomas. In future, small molecule TOPK inhibitors should be developed to enhance lung cancer treatment efficacy with EGFR-TKIs.

## MATERIALS AND METHODS

### Reagents

Active TOPK was purchased from Upstate Biotechnology (Charlottesville, VA, US). Antibodies against PBK/TOPK, phospho-PBK/TOPK (Thr7), c-Jun and CDC2 were purchased from Abcam Corporation (Cambridge, MA, US). Antibodies against TOPK, EGFR, phospho-EGFR (Tyr1068), phospho-c-Jun (Ser63), phospho-c-Jun (Ser73), ERK1/2, phospho-ERK (Thr202/Tyr204), JNK, phospho-JNK (Thr183/Tyr185) and cyclin D1 were purchased from Cell Signaling Technology (Beverly, MA, US). The JetPEI reagent was purchased from Qbiogene (Montreal, Quebec, Canada) and EGF from BD Biosciences (San Jose, CA, US).

### Immunofluorescent staining

The lung adenocarcinoma tissue array slide was blocked with 5% donkey serum albumin in 600 μ1 phosphate-buffered saline/0.03% Triton X-100, (pH 6.0) in a humidified chamber for 1 h at room temperature and then were immunostained with 1:100 anti-TOPK raised in mouse (Santa Cruz Biotechnology) and 1:200 donkey anti-mouse IgG conjugated to Cy2 (Jackson ImmunoResearch Laboratories). Image stacks were captured (×20) at room temperature using laser scanning confocal microscopy (NIKON Clsi Confocal Spectral Imaging System; NIKON Instruments Co., Melville, NY). The intensity of immunofluorescence was scored using EZ-C1 3.90 FreeViewer (NIKON).

### Cell culture and transfection

The human lung cancer cell lines A549, H358, H460, H520, H441, H1650, H1975 and Calu-3 were purchased from ATCC (Manassas, VA, USA). The human embryonic kidney (HEK) 293T cell line was obtained from the Cell Bank of Shanghai Institute for Biological Sciences, Chinese Academy of Sciences. All cell lines were characterized by gene profiling by the providers, and were propagated and frozen for future study after receipt. Each vial of frozen cells was thawed and maintained in culture for a maximum of 8 weeks. Enough frozen vials were available for each cell line to ensure that all cell-based experiments were conducted on cells that had been tested and in culture for 8 weeks or less.

Lung cancer cells were cultured in RPMI 1640 or DMEM medium supplemented with 10% fetal bovine serum (FBS) and maintained at 37°C in a humidified incubator with 5% CO_2_. The cultures were split at 90% confluence, and the media were changed every 2 days. When cultures reached 50–60% confluence, transfection was performed using JetPEI following the manufacturer's suggested protocol. The cells were cultured for 36–48 h, and then proteins were extracted for further analysis.

### Cell proliferation and cytotoxicity assay

For cell proliferation assay, cells (500 /well) were seeded into 96-well plates and incubated at 37°C with 5% CO_2_. After incubation for 24h, 48h or 72h, 10 μL of CCK-8 solution (Shanghai R&S Biotechnology Co., Ltd) was added and then incubated for 1 hour at 37°C with 5% CO_2_. Absorbance was measured at 450 nm in a plate reader. For cytotoxicity assay, cells were seeded into 96-well plates at an initial density of 5×10^3^ cells /well and cultured for 12 h, and then stimulated with increased concentrations of gefitinib for additional 48 h. Cell viability was assessed using CCK-8 solution and absorbance was measured at 450 nm.

### Tissue array

A human lung cancer tissue array (U.S. Biomax, Rockville, MD) was prepared and analyzed according to the protocol provided by the manufacturer. The samples were blocked in 5% goat serum albumin in 600 μL 1×phosphate-buffered saline (PBS) supplemented with 0.03% Triton X-100 (pH 6.0) in a humidified chamber for 1 h at room temperature and then incubated with TOPK antibody (1:100 dilution in 500 μL 1×PBS/0.03% Triton X-100 (pH 6.0)) at 4°C in a humidified chamber overnight. The slides were washed and hybridized for 2 h with secondary antibody conjugated to Cy2 (Jackson ImmunoResearch Laboratories, West Grove, PA) (1:200 dilution) at room temperature in the dark. Slides were washed in PBS (2×5 min). The expression of TOPK was observed by laser scanning confocal microscopy (NIKON C1si Confocal Spectral Imaging System; NIKON Instruments Co., Melville, NY).

### Lentiviral infection

The lentiviral expression vectors, including Gipz-shTOPK, and packaging vectors, including pMD2.0G and psPAX, were purchased from Addgene (Cambridge, MA). To prepare TOPK viral particles, each viral vector and packaging vectors (pMD2.0G and psPAX) were transfected into 293T cells using JetPEI following the manufacturer's suggested protocols. The transfection medium was replaced 4 h after transfection and then cells were cultured for 36 h. The viral particles were harvested, filtered using a 0.45-μm syringe filter, combined with 8 μg/mL polybrene (Millipore, Boston, MA), added to 60% confluent A549 lung cancer cells, and incubated overnight. The cell culture medium was replaced with fresh complete growth medium for 24 h and then cells were selected with puromycin (8.0 μg/mL) for 36 h. The selected cells were used in experiments.

### Anchorage-independent cell growth

A549 cells (8×10^3^ per well of 6-well plate) suspended in DMEM supplemented with 10% FBS were added to 0.3% agar containing different concentrations of gefitinib (top layer) over a base layer of 0.6% agar. These soft-agar cultures were maintained at 37°C in 5% CO_2_ for 3 weeks and then colonies were counted under a microscope using the Image-Pro Plus software (v.4) program (Media Cybernetics).

### Modeling studies

According to previous reports [52-54], the 3D structures of the TOPK (accession number: Q96KB5) and c-Jun (accession number: NP_002219) proteins were built using the MODELER module in the InsightII 2005 software package [54, 55]. The protein structures generated were adequately equilibrated by MD simulation using the GROMACS4.5.5 program [56] and Charmm27 force field [57], as recommended previously [54, 58]. The docking simulations were performed using the ZDOCK and RDOCK modules within InsightII 2005 [59-61]. The optimally docked complex was selected on the basis of energy and size of cluster, and then optimized using the conjugated gradient algorithm with a convergence criterion of 0.01 kcal·mol-1·Å-1. The energy-minimized docked complex was sufficiently equilibrated by 50.0 ns MD simulations using the GROMACS4.5.5 program [56] and Charmm27 force field [57]. Details of the MD simulation setup are consistent with previously published work [58].

### *In vitro* kinase assay

To detect γ-^32^P incorporation, 2 μg wild-type His-c-Jun and mutant c-Jun were mixed separately with active TOPK (0.2 μg/50-μL reaction; Cell Signaling, Danver, MA) in 5× kinase buffer containing 10 μM unlabeled ATP and 10 μCi [γ-^32^P] ATP (New England Biolabs, Ipswich, MA) and incubated at 30°C for 30 min. The reaction was stopped by the addition of 6× SDS loading buffer. Samples were separated by 10% SDS-PAGE and γ-^32^P-labeled wild-type or mutant c-Jun was visualized by autoradiography or Coomassie blue staining.

### AP-1 activity assay

HEK293 cells stably transfected with an AP-1 luciferase reporter plasmid were transfected with pcDNA4-wt-c-jun or pcDNA3.1A-TOPK plasmid, and then the cells were further transiently transfected with the PRL-SV40 plasmid (Promega, Madison, WI). Cells were starved in 0.1% FBS-DMEM for 24 h, after which they were stimulated with EGF (10 ng/mL) for 12 h. Then, the cells were disrupted with lysis buffer and luciferase activity was measured using a luminometer (Monolight 2010, San Diego, CA).

### Quantitative RT-PCR

Total RNA was extracted from cells using the TRIzol reagent (Invitrogen/Life Technologies) according to the manufacturer's protocol. Reverse transcription was performed using SuperScript^TM^ II reverse transcriptase (Invitrogen/Life Technologies), and cDNAs were amplified and detected using the SYBR Premix Ex Taq^TM^ (TaKaRa, Dalian, China). The primers used for PCR were as follows: 5′-GATCATTGCTCCTCCTGAGC-3′ and 5′-ACTCCTGCTTGCTGATCCAC-3′ for β-actin, 5′-TGTGCATCTACACCGACAAC-3′ and 5′-AGGAAGTGTTCAATGAAATCGT-3′for *CCND1*, and 5′-AAAATTGGAGAAGGTACCTATGGA-3′and 5′-CCCTTCCTCTTCACTTTCTAGTCTG-3′ for *CDC2*.

### Western blotting

Cells were harvested, and proteins were extracted, separated on an SDS/PAGE gel, transferred onto PVDF membranes and subjected to immunoblot analyses. Blotting was performed using antibodies targeting TOPK (1:500), phosphorylated TOPK (pTOPK, 1:500), c-Jun (1:500), p-c-Jun (Ser63, 1:500), p-c-Jun (Ser73, 1:500), ERK (1:500), pERK (1:500), JNK (1:500), pJNK (1:500), cyclin D1 (1:250), Cdc2 (1:500) and β-actin (1:1000, Sigma-Aldrich, St. Louis, MO).

### Immunoprecipitation

Cells were lysed in buffer containing 1% (v/v) Nonidet P-40, 0.5 mM EGTA, 5 mM sodium orthovanadate, 10% (v/v) glycerol, 100 μg/mL phenylmethylsulfonyl fluoride, 1 μg/mL leupeptin, 1 μg/mL pepstatin A, 1 μg/mL aprotinin and 50 mM HEPES, pH 7.5. Aliquots of 500 μL diluted lysate (1 μg protein/μL) were incubated overnight with 5 μL antibody against TOPK or c-Jun. The immune complex was captured by adding 80 μL of a 1:1 (v/v) protein A-Sepharose 4B bead suspension and incubating the mixture for an additional 90 min. The beads were harvested and the proteins bound to them were resolved by SDS-PAGE and analyzed by Western blot. The primary antibodies used for immunoprecipitation are described in the Western blotting methods.

### *In vivo* tumor xenograft mouse model

BALB/c nude mice (Institute of Zoology, Chinese Academy of Sciences) that were 4–6 weeks old were challenged subcutaneously with lung cancer A549 cells modified with a control lentiviral construct (A549shmock) and those modified to express TOPK-targeted shRNA (A549shTOPK) (1×10^7^) to allow tumor development. When palpable tumors (approximately 100 mm^3^) arose, the mice in each group were randomly allocated to treatment groups of five animals: (1) no treatment (control) or (2) gefitinib (25 mg/kg/d) by oral gavage. Tumor growth was monitored by caliper measurements of the two perpendicular diameters every 3 days, and the volume of the tumor was calculated with the formula V = (width^2^×length/2). All procedures were performed in compliance with the Regulations for the Administration of Affairs Concerning Experimental Animals (approved by the State Council of the People's Republic of China) and were approved by the Experimental Animal Ethics Committee of Fourth Military Medical University.

### Statistical analysis

Statistical significance was assessed by comparing mean (± SD) values with Student's *t*-test for independent groups. *P* ≤ 0.05 was considered statistically significant.

## SUPPLEMENTARY MATERIAL FIGURES AND TABLE


