# Supplementary material for: TOPK promotes lung cancer resistance to EGFR tyrosine kinase inhibitors by phosphorylating and activating c-Jun
Source: Oncotarget. 2016 Jan 6;7(6):6748–64. doi: 10.18632/oncotarget.6826 (PMC4872746; doi:10.18632/oncotarget.6826)
Supplement: Supplementary file 1 [file oncotarget-07-6748-s001.pdf]

TOPK promotes lung cancer resistance to EGFR tyrosine kinase inhibitors by phosphorylating and activating c-Jun

Supplementary Material

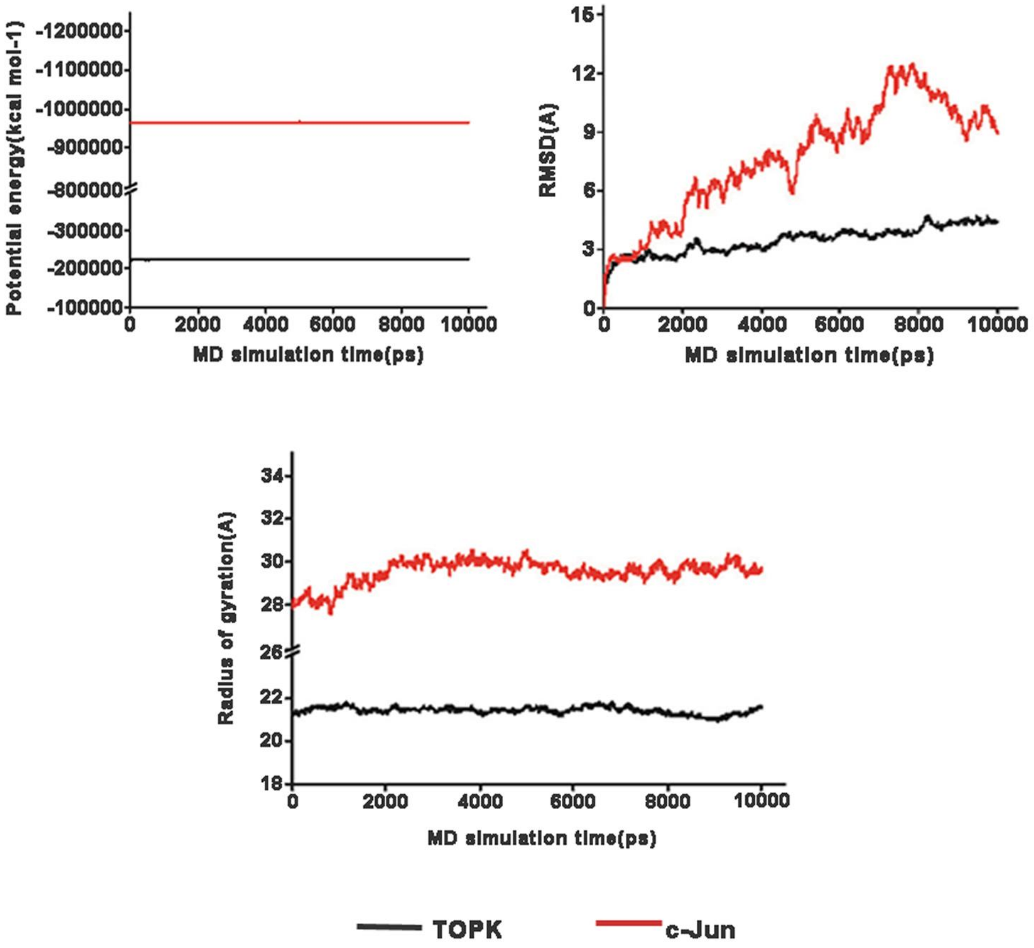

Supplemental Figure S1. Potential energy, backbone-atom RMSD and radius of gyration for TOPK (in black) and c-Jun (in red) proteins with respect to simulation time.

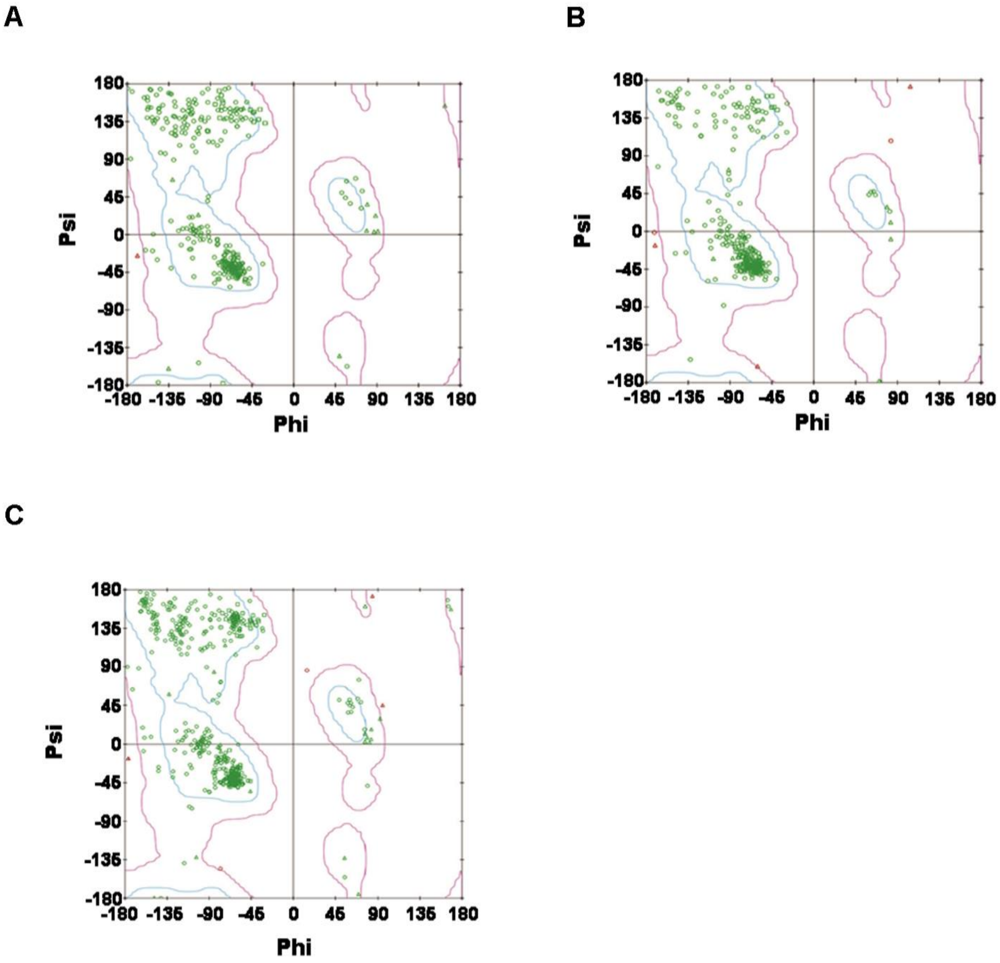

Supplemental Figure S2. Ramachandran plots of TOPK (A), c-Jun (B) and TOPK-c-Jun (C) after MD simulations. The Ramachandran plot shows the phi-psi torsion angles of all residues in the structure. The coloring/shading on the plot represents the different regions: the darkest areas (shown here in red) correspond to the "core" regions representing the most favorable combinations of phi-psi values. The percentage of residues in the "core" regions is one of the best indications of stereochemical quality.

Supplemental Table 1. Hydrogen bonds formed between TOPK and c-Jun

Receptor residue	Ligand residue	Interaction constituents (Donor→ Acceptor)	Distance (Å)	Angle (°)
TOPK:Arg130	c-Jun:Asn85	Arg130:HH22 - Asn85:OD1	1.74	162.9
TOPK:Phe179	c-Jun:Glu77	Phe179:HN - Glu77:OE2	1.68	158.8
TOPK:Glu180	c-Jun:Glu77	Glu180:HN - Glu77:OE2	1.85	137.7
TOPK:Lys183	c-Jun:Tyr10	Lys183:HZ1 - Tyr10:OH	2.48	111.4
TOPK:Glu116	c-Jun:Tyr10	Tyr10:HH - Glu116:OE1	2.39	124.5
TOPK:Asp321	c-Jun:Tyr28	Tyr28:HH - Asp321:O	1.77	138.5
TOPK:Asp321	c-Jun:Tyr28	Tyr28:HH - Asp321:OXT	2.13	152.2
TOPK:Asp321	c-Jun:Lys32	Lys32:HZ2 - Asp321:OD2	1.67	155.3
TOPK:Thr320	c-Jun:Lys32	Lys32:HZ3 – Thr320:O	1.77	149.5
TOPK:Glu180	c-Jun:Lys35	Lys35:HZ1 - Glu180:OE2	1.56	166.1
TOPK:Asp321	c-Jun:Lys35	Lys35:HZ3 - Asp321:OXT	1.63	162.0
TOPK:Leu56	c-Jun:Lys50	Lys50:HZ1 - Leu56:O	2.46	106.5
TOPK:Ser57	c-Jun:Lys50	Lys50:HZ1 - Ser57:O	1.80	143.6
TOPK:Ser52	c-Jun:Lys50	Lys50:HZ3 - Ser52:OG	2.12	135.6
TOPK:Val174	c-Jun:Lys70	Lys70:HZ1 – Val174:O	1.82	153.6
TOPK:Asp178	c-Jun:Ala72	Ala72:HN – Asp178:OD1	1.65	175.5
TOPK:Asp178	c-Jun:Ser73	Ser73:HN - Asp178:OD1	1.87	163.3
